# Update on Babesiosis

**DOI:** 10.1155/2009/984568

**Published:** 2009-08-27

**Authors:** Edouard Vannier, Peter J. Krause

**Affiliations:** ^1^Division of Geographic Medicine and Infectious Diseases, Tufts Medical Center, 800 Washington Street, Box 041, Boston, MA 02111, USA; ^2^Department of Epidemiology and Public Health, Yale School of Medicine, 60 College Street, Room 600, P.O. Box 208034, New Haven, CT 06520, USA

## Abstract

Human babesiosis is an emerging tick-borne infectious disease caused by intraerythrocytic protozoan species of the genus *Babesia* with many clinical features similar to those of malaria. Over the last 50 years, the epidemiology of human babesiosis has changed from a few isolated cases to the establishment of endemic areas in the northeastern and midwestern United States. Episodic cases are reported in Europe, Asia, Africa, and South America. The severity of infection ranges from asymptomatic infection to fulminant disease resulting in death, although the majority of healthy adults experience a mild-to-moderate illness. People over the age of 50 years and immunocompromised individuals are at the highest risk of severe disease, including those with malignancy, HIV, lacking a spleen, or receiving immunosuppressive drugs. Asymptomatic carriers present a blood safety risk when they donate blood. Definitive diagnosis of babesial infection generally is made by microscopic identification of the organism on thin blood smear, amplification of *Babesia* DNA using PCR, and detection of *Babesia* antibody in acute and convalescent sera. Specific antimicrobial therapy consists of atovaquone and azithromycin or clindamycin and quinine. Exchange transfusion is used in severe cases. The use of multiple prevention strategies is recommended and consists of personal, residential, and community approaches.

## 1. Introduction

Human babesiosis is an emerging tick-borne infectious disease caused by an intraerythrocytic protozoan and has many clinical features similar to those of malaria. The parasite was first described in cattle by Babes in 1888 [1]. The first human case was described in 1957 [2]. Over the past 50 years, the epidemiology of the disease has changed from a few isolated cases to the establishment of endemic areas in southern New England, New York, New Jersey, and the northern Midwest [3–8]. Isolated cases are reported over a wide geographic range in Europe, Asia, Africa, and South America [9–17]. We will review the epidemiology, clinical characteristics, diagnosis, and treatment of infections caused by the four *Babesia* species that most commonly infect people: *B. microti*, *B. duncani*, *B. divergens*, and *B. venatorum*.

## 2. Epidemiology

### 2.1. United States

#### 2.1.1. *B. microti* Infection

The most common cause of human babesiosis is *Babesia microti*, a *Babesia* of mice and other small rodents that is endemic in the United States [5–8]. Most cases occur along the northeastern seaboard. The first confirmed case was a normosplenic individual on Nantucket Island off the coast of Massachusetts [18]. Additional cases were soon identified and the disease became known as Nantucket fever. Other islands off the coast of southern New England subsequently became recognized as endemic areas, including Martha's Vineyard, the Elizabeth Islands, Block Island, Shelter Island, the eastern part of Long Island, and Fire Island [19]. Endemic areas on the mainland include Cape Cod, the southwestern counties of Rhode Island, and southeastern Connecticut [6, 19, 20]. Babesiosis has been diagnosed in individuals from the Lower Hudson Valley (Westchester, Putnam, Dutchess, and Columbia counties) in New York State, and is endemic in New Jersey [21–23]. *B. microti* is also the etiologic agent of babesiosis in the northern midwestern region of the United States, particularly in Wisconsin and Minnesota [24–26]. A few isolated cases have been noted in Indiana [27, 28]. Human babesiosis due to *B. microti* recently has been reported from Europe while *B. microti*-like species have been described as the cause of infection in people living in Asia [13, 17, 29]. 

The distribution of *B. microti* overlaps with that of *Borrelia burgdorferi*, the agent of Lyme disease, as both organisms are transmitted by *Ixodes scapularis* in the United States [19, 30]. Although the geographic distribution of babesiosis appears to be restricted to certain foci within the areas of endemicity for *B. burgdorferi*, the endemic range is expanding. For this reason and because the number of reported cases has dramatically increased over the last two decades, babesiosis is recognized as an emerging infectious disease [8, 31, 32]. The increase in incidence has often been attributed to the expansion of the deer population because these vertebrates are the primary host for adult ticks, although they are not competent reservoirs for *B. microti*. Other factors include better awareness of the disease by local physicians, an encroachment of humans on wildlife habitat, and increasing travel of immunosuppressed individuals to endemic areas.

Although most *B. microti* infections are acquired by tick bites from May through October, at least 70 patients in the United States have acquired babesiosis by transfusion of contaminated blood products from asymptomatic donors [33–36]. Blood products typically are fresh or frozen packed red blood cells, although one case has been attributed to platelet transfusion. Several cases of neonatal babesiosis have been reported, and these were acquired by tick bite, transfusion, or transplacental transmission [37, 38]. 

#### 2.1.2. *B. duncani* Infection

*B. duncani* is the name given to the previously designated WA1 *Babesia* [3]. All nine documented cases of *B. duncani* infection have occurred on the west coast of the United States. Two people acquired the babesial infection by transfusion of blood products while the remaining cases were attributed to tick bites [39, 40]. One blood donor was asymptomatic at time of donation whereas the other reported a 10-day episode of nausea. Both recipients were immunologically at risk; one was an elderly man and the other was a premature infant. Among the five other symptomatic cases, one was a young normosplenic adult living in Washington State (index case) whereas four were splenectomized and lived in California [41, 42]. One of these four men died. Sequence analysis of the entire 18S rRNA gene indicated that the organisms causing babesial illness in the index case from Washington State and in the two cases acquired by blood transfusion were phylogenetically indistinguishable whereas the organisms acquired by three of the four splenectomized cases in California formed a separate but closely related phylogenetic group [3, 43]. The seroprevalence of *B. duncani* infection has been found to vary from 4% to 17% [41].

#### 2.1.3. *B. divergens*-Like Infection

*B. divergens*-like organisms have been identified in three cases, two from the Midwest (Missouri and Kentucky) and one from Washington State [4, 44, 45]. All three patients had risk factors for severe babesial disease, namely, age greater than 50 years and splenectomy. Sequence analysis of the entire 18S rRNA gene indicated that the Missouri isolate (MO1) and the Kentucky isolate (KY) were identical to each other and to piroplasms found in eastern cottontail rabbits on Nantucket Island [46]. These isolates differed by a few bp in their 18S rRNA gene from the isolate of the Washington State patient and from the *B. divergens* organisms of European cattle. Because the piroplasms isolated from eastern cottontail rabbits are not infectious to cattle, the isolates obtained from the three US patients are now referred to as *B. divergens*-like organisms.

### 2.2. Europe

#### 2.2.1. *B. divergens* Infection

About 40 cases of *B. divergens* infection have been documented in Europe, mostly from countries with extensive cattle industry such as France, Ireland, and Great Britain [47–49]. Cases have sporadically been reported from Sweden, Switzerland, Spain, Portugal, and Croatia (index case). Nearly all patients were splenectomized prior to the onset of *Babesia* infection. Because cattle are the reservoir for *B. divergens*, persons at risk of contracting babesiosis are farmers or people vacationing in rural areas. Limited data suggest that infection with *B. divergens* can be asymptomatic in normosplenic individuals [47]. There are no reports of *B. divergens* infection acquired by blood transfusion [49].

#### 2.2.2. *B. venatorum* (EU1) Infection

Because the advent of PCR allows for molecular characterization of babesial organisms, a new *Babesia* species has been identified in Europe. Originally referred to as EU1, the name “*B. venatorum*” was later proposed [12]. *B. venatorum*, together with *B. odocoilei* that infects white-tail deer in the United States, form a sister group to that of *B. divergens*. Three cases of infection with *B. venatorum* have been documented [12, 50]. All three were men beyond 50 years of age who had been splenectomized. Unlike *B. divergens* infections that are fulminant and often fatal, *B. venatorum* infections have varied from mild to severe but have not been fatal.

## 3. Clinical Characteristics

### 3.1. *B. microti* Infection

The severity of *B. microti* infection is variable, depending primarily on the immune status of the host. Several clinical syndromes have been described, including asymptomatic infection, mild-to-moderate viral-like illness, and severe disease with a fulminant course that sometimes results in death or a persistent relapsing illness. Concurrent infection with Lyme disease increases neither the number nor the duration of symptoms of babesiosis [30, 51]. It is unclear whether this is also true of concurrent babesiosis and human granulocytic anaplasmosis.

#### 3.1.1. Asymptomatic Infection

Following transmission of the babesial parasite, the incubation period may last from 1 to 9 weeks [5, 8]. Many people who are infected with *B. microti* never experience symptoms, as suggested by the disparity between seroprevalence and the number of reported cases. The frequency of asymptomatic *B. microti* infection was derived from an epidemiologic study of babesiosis carried out on Block Island, Rhode Island [6]. About a third of babesial infections at this site were asymptomatic, including 19% (13 of 67) of adults and 40% (4 of 10) of children [6]. Asymptomatic infection may persist for months or years following resolution of symptomatic babesiosis [36]. It is uncertain whether patients experiencing asymptomatic babesial infection are at risk for any long-term complications. They may transmit the infection if they donate blood [33].

#### 3.1.2. Mild-to-Moderate Illness

Most cases of *B. microti* infection consist of a mild to moderate viral-like illness. These cases typically begin with a gradual onset of malaise and fatigue followed by intermittent fever and one or more of the following: chills, sweats, headache, arthralgia, myalgia, anorexia, cough, and nausea (Table 1) [30, 52–54]. Less commonly noted are gastrointestinal symptoms, weight loss, conjunctival injection, and emotional lability [53–57]. On physical examination, fever is commonly observed and pallor, mild splenomegaly or hepatomegaly occasionally may be noted. The illness usually lasts for a week to months, occasionally with prolonged recovery that can last more than a year [30, 36, 53, 56, 57]. Parasitemia may continue even after the patient feels well and rarely may persist for more than two years after the initial episode [36]. 

#### 3.1.3. Severe Disease

Severe disease generally occurs in people with underlying immunosuppressive conditions that include HIV, malignancy, immunosuppressive medication, and splenectomy [50, 56, 58–60]. On physical examination, jaundice, retinal infarcts, or ecchymoses and petechiae may be noted [55, 58, 61]. The most common complications of severe babesiosis include acute respiratory failure, congestive heart failure, DIC, liver and renal failure, and splenic rupture [52]. A mortality rate of 5% was noted in a retrospective study of 136 patients experiencing *B. microti* infection on Long Island, New York, only one of whom was known to be immunocompromised [7]. In another study, the mortality rate among patients hospitalized for babesiosis was 9% [52]. The mortality rate is even higher among immunocompromised hosts. In a recent case-control study of 14 patients who were highly immunocompromised, 21% died and the remainder experienced a prolonged, relapsing course of illness, sometimes lasting more than a year, despite multiple courses of standard antibabesial therapy [59]. People 50 years of age and older also are more likely to experience severe babesiosis [52, 54, 62].

### 3.2. *B. duncani* Infection

Although studies of infection in hamsters suggest that *B. duncani* is more pathogenic than *B. microti*, the small number of reported *B. duncani* cases does not allow firm conclusions [32]. Of the nine reported cases of *B. duncani*, one died, one experienced pulmonary edema and renal insufficiency, and the remainder had a relatively mild clinical course or were asymptomatic [3, 39, 40, 42, 63]. Parasitemia ranged from 1% to 54%. Symptoms of *B. duncani* are similar to those of *B. microti* and consist of fever, chills, headache, sweats, myalgia, nausea, vomiting, diarrhea, dark urine, and fatigue.

### 3.3. *B. divergens* Infection

Nearly all 40 cases of *B. divergens* infection reported in Europe had been splenectomized and suffered a severe form of babesiosis [47–49]. Signs and symptoms begin 1 to 3 weeks after tick transmission and consist of high fever (40-41°C) with severe intravascular hemolysis that results in hemoglobinemia, hemoglobinuria, and jaundice. Headache, shaking chills, intense sweating, myalgia, and abdominal pain are common. More than half the cases experienced a rapid onset of renal failure and pulmonary edema. Ecchymoses, petechiae, congestive heart failure, and coma also have been reported. The illness generally is fulminant, lasting about a week and ending in death in more than a third of patients or in a prolonged convalescence.

### 3.4. *B. venatorum* (EU1) Infection

All three reported cases of *B. venatorum* infection had a history of Hodgkin's disease and had been splenectomized [12, 50]. One experienced mild babesiosis, whereas two had moderate-to-severe illness. In these two patients, babesiosis was concurrent with a relapse of nodular lymphocyte-predominant Hodgkin's lymphoma or with stage IIIA diffuse large B cell lymphoma. All three patients were admitted to the hospital and recovered after antibabesial therapy. One had a prolonged relapsing illness that eventually cleared. Peak parasitemia ranged from 1% to 30%. Symptoms included fever, dark urine, fatigue, chills, headache, confusion, jaundice, sweats, and shortness of breath.

## 4. Diagnosis

The diagnosis of babesiosis should be considered in patients who live or travel in areas that are endemic for babesiosis and who experience a viral-like illness in the late spring, summer, or autumn (Table 2). As the symptoms and signs are relatively nonspecific, laboratory testing is required for diagnosis. In some cases, physicians may choose to obtain screening laboratory testing such as a CBC, platelet count, and liver enzymes before ordering specific diagnostic tests. Most cases of babesiosis are accompanied by some degree of hemolytic anemia with an elevated reticulocyte count. Thrombocytopenia is common. The leukocyte count usually is normal to slightly decreased. Elevated serum liver enzymes occur in about half the patients. Proteinuria and an elevated blood urea nitrogen and serum creatinine also may be noted. Specific laboratory tests that are used for the diagnosis of babesiosis are described below. Specific tests for concurrent Lyme disease and human granulocytic anaplasmosis should be performed in patients presenting with clinical manifestations that suggest coinfection, such as an erythema migrans type rash or neutropenia.

### 4.1. Microscopic Identification

**Figure 1:**
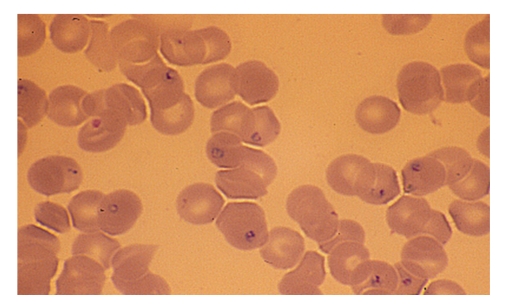
Ring forms of *B. microti* revealed by Giemsa staining of a human blood film (magnification 1000 x).

Specific diagnosis of babesiosis can be made by microscopic identification of the organism using Giemsa stains of thin blood smears [64, Figure 1]. Thick blood smears also may be performed but the babesial organisms are small and may be difficult to visualize. *B. microti* and other *Babesia* spp. are round, oval, or pear-shaped and have a blue cytoplasm with a red chromatin. The ring form is most common and can be mistaken for early stage ring forms of *Plasmodium falciparum*. Multiple thin blood smears should be examined when only a few erythrocytes are infected, particularly in the early stage of illness when most people seek medical attention. PCR and serology should be performed if only a few ring-like structures are observed. Parasitemia seldom exceeds 5% in normal hosts but may be as high as 85% in asplenic individuals [55]. *B. divergens* and related organisms often appear as paired piriforms in human red blood cells. Pairs of *B. divergens* and *B. divergens*-like organisms are located in a subcentral, central, or peripheral position [46]. *B. venatorum* are morphologically indistinguishable from *B. divergens* [12, 50]. The tetrad form, referred to as a Maltese cross, is pathognomonic of small *Babesia* spp. such as *B. microti* and *B. duncani* [3]. *B. duncani* display more tetrad forms than *B. microti*.

### 4.2. Polymerase Chain Reaction

The polymerase chain reaction (PCR) provides a highly sensitive and specific test for detecting *Babesia* DNA in blood and identifies the *Babesia* species [65, 66]. Rigorous precautions are required to avoid false positive results. PCR is recommended when parasites are not identified on blood smears but symptoms and history are suggestive of babesiosis. As with all *Babesia* specific tests, it should only be performed in laboratories that are experienced in such testing and meet the highest laboratory performance standards.

### 4.3. Serology

Babesial infection can be confirmed by serologic testing using the indirect immunofluorescent assay (IFA) [67, 68]. During the acute phase of *B. microti* illness, IgG titers usually exceed 1 : 1024 and decline to 1 : 64 or less within 8 to 12 months. Thus, IgG titers of 1 : 1024 or greater usually signify active or recent infection [69]. The detection of IgM is indicative of recent infection [67]. Although seroconversion occurs in virtually all immunocompetent individuals infected with *B. microti*, the diagnosis of active babesial infection based on serologic findings alone is suspect. Serology usually is not considered in cases attributed to *B. divergens* because the illness becomes fulminant before antibody can be detected. Sera from patients infected with *B. divergens*-like organisms or *B. venatorum* cross-react with antigen from *B. divergens* [41, 42, 63]. Sera from patients infected with *B. duncani* or related organisms do not cross-react with *B. microti* antigen [41, 42]. Sera from patients infected with one of several *Babesia* species may cross-react with antigen from *Plasmodium* species, but titers are almost always low (1 : 16 or lower).

### 4.4. Amplification of *Babesia* in Laboratory Animals

*Babesia* parasitemia can also be detected by injecting patient blood by the intravenous or intraperitoneal route into such laboratory animals as hamsters or gerbils [65]. This test is only available in a few research laboratories and is seldom useful for diagnosis. *B. microti* is easily detected in hamsters whereas *B. duncani* is often lethal in these animals [32]. This approach is not suited for rapid diagnosis, as *Babesia* usually do not appear in the blood of the laboratory animal until two to four weeks after inoculation.

## 5. Treatment

### 5.1. *B. microti* Infection

#### 5.1.1. Mild-to-Moderate Disease

Most people infected with *B. microti* experience mild-to-moderate disease and can be successfully treated with a combination of atovaquone and azithromycin administered for 7 to 10 days [70, 71]. This combination was shown to be as effective as clindamycin and quinine, the first drug combination used in the treatment of babesiosis. The two regimens were directly compared in adults in a prospective, nonblinded randomized control trial [71]. While these drug combinations were similarly effective in clearing parasitemia and achieving resolution of symptoms, adverse effects were reported in 15% of subjects who received atovaquone and azithromycin compared with 72% of subjects who received clindamycin and quinine. The most common side effects associated with atovaquone and azithromycin were diarrhea and rash while those of clindamycin and quinine were tinnitus, hearing loss, and diarrhea. About a third of the subjects receiving clindamycin and quinine suffered from adverse reactions that were severe enough to require a decrease in dosage or discontinuing the medication. Only 2% of subjects taking atovaquone and azithromycin experienced such severe drug reactions. Although the atovaquone and azithromycin combination has not been studied in a controlled trial for pediatric use, cure has been achieved with use of this regimen in a child [72]. Clindamycin and quinine should be substituted for atovaquone and azithromycin when patients do not respond well to atovaquone and azithromycin.

#### 5.1.2. Severe Disease

The combination of clindamycin (administered intravenously) and quinine given for 7 to 10 days is the treatment of choice for severe babesiosis. (Table 3) [70, 73]. Exchange red blood cell transfusion is indicated for all babesiosis patients experiencing heavy parasitemia (≥10%) or who have significant pulmonary, renal, or hepatic compromise [74–77]. Partial or complete exchange transfusion rapidly decreases parasite burden and removes toxic byproducts of *Babesia* infection.

#### 5.1.3. Persistent Relapsing Disease

Babesial infection may persist and symptoms relapse in people with significant underlying immunosuppression despite standard combination antimicrobial therapy [59, 77]. Atovaquone-proguanil (250 mg–100 mg) was used to eradicate parasitemia in one such patient [77]. In a recent case-control study of chronic babesiosis in 14 highly immunocompromised patients, no single antimicrobial combination was uniformly superior to another in achieving resolution of infection [59]. Rather, cure was associated with duration of therapy for a minimum of six weeks and for at least two weeks after the last positive blood smear. Interestingly, the majority of case patients in the study had underlying B-cell lymphoma and had been treated with the anti-CD20 monoclonal antibody (Rituximab) prior to acute babesial infection, thereby dramatically impairing B-cell activity. It is important to recognize that a 7-to-10 day course of antibabesial therapy is sufficient for many immunocompromised patients. Thus, when acute babesiosis responds to a standard therapeutic course with resolution of symptoms and clearance of parasitemia on blood smear, no further treatment is required. Because immunosuppressed individuals are at increased risk for relapsing babesiosis, close clinical follow-up with repeat blood smears, *Babesia* PCR, and complete blood counts should be performed. 

#### 5.1.4. Asymptomatic Infection

In the unusual event that patients are identified with asymptomatic babesial infection, no treatment should be given unless parasitemia, as determined by blood smear or PCR, persists for longer than three months [70]. In this case, a one-week course of atovaquone and azithromycin should be considered. People who have positive serology, but negative blood smears and negative PCR should not be treated, as they likely have resolved the infection. Immunocompromised patients experiencing persistent asymptomatic parasitemia should have blood smears performed every month or two until they clear the infection.

### 5.2. *B. duncani* Infection

Recommended treatment consists of clindamycin and quinine [3, 39, 40, 42, 63]. Red cell exchange transfusion should be considered in the treatment of severe cases [41]. Renal insufficiency may require hemodialysis.

### 5.3. *B. divergens* and *B. divergens*-Like Infections

The morbidity and mortality rate of *Babesia divergens* infections are high and any such case should be considered a medical emergency [47, 49]. It is recommended that these infections be treated with exchange transfusion and clindamycin and quinine. One mild case of *B. divergens* infection was successfully treated with the combination of pentamidine and trimethoprim-sulfamethoxazole whereas another only required an abbreviated course of clindamycin and quinine (the quinine was stopped after 4 days) [79, 80]. In both cases, exchange transfusion was not needed. The *Babesia divergens*-like infections reported from Kentucky and Washington State were successfully treated with clindamycin and quinine or quinidine, but the case from Missouri died despite clindamycin and quinine therapy [4, 44, 45].

### 5.4. *B. venatorum* Infection

Of the three cases of *B. venatorum* infection, one cleared on clindamycin alone [12, 50]. The other two were given clindamycin and quinine. One of these patients was highly immunocompromised and relapsed after clindamycin and quinine was discontinued. He required a prolonged course of atovaquone and azithromycin followed by atovaquone alone for cure. In the third case, blood transfusion was given for massive hemolysis, and the infection eventually cleared following antimicrobial therapy.

## 6. Prevention

The use of multiple strategies is most likely to be effective for prevention of babesiosis. These include personal, residential, and community approaches [81–88]. One obvious personal protective measure is to avoid areas where ticks, mice, and deer are known to thrive, especially from May through October [84]. Immunocompromised people who are at increased risk of severe babesiosis need to avoid such areas. Anyone who may contact foliage in endemic sites should wear clothing that covers the lower part of the body, tuck cuffs of the trousers into stockings, and spray or impregnate clothing with permethrin (Permanone) [86]. DEET-containing products should be applied to the skin if legs remain uncovered. After travel through a high-risk area, a tick check should be performed and attached ticks should be removed as soon as possible by use of tweezers [84]. No data is available on the use of prophylactic antimicrobials after a tick bite to prevent babesiosis nor has a human babesiosis vaccine been developed.

Property modifications can help reduce tick exposure. These include keeping grass mowed, removing leaf litter at the edge of lawns, sealing stonewalls to decrease the number of mice, using plantings that do not attract deer, and fencing to keep deer away [84]. Acaracides can be applied to property, rodents (Damminix or fipronil), or deer (four poster device) [82, 84, 88]. Elimination of the deer population may sharply reduce the risk of babesiosis and other tick-borne infections. Within 3–5 years after deer were eliminated on Great Island off Cape Cod, Massachusetts, the density of *I. scapularis* ticks fell precipitously [87]. Currently, the Red Cross and other blood donation agencies prohibit people with a history of babesiosis from donating blood in order to prevent transfusion-related cases. Additional measures such as screening blood donors for silent *Babesia* infection or inactivation of *Babesia* parasites in units of blood prior to transfusion have been considered but are not yet in use [89, 90]. 

## Figures and Tables

**Table 1:** The most common symptoms of babesiosis caused by *Babesia microti* infection.

Symptom	Percentage of outpatients (*n* = 41)	Percentage of inpatients (*n* = 173)	Percentage of all patients (*n* = 214)
Fever	68	89	85
Fatigue	78	79	79
Chills	39	68	63
Sweats	41	56	53
Headache	75	32	39
Myalgia	37	32	33
Anorexia	25	24	24
Cough	17	23	22
Arthralgia	31	17	18
Nausea	22	9	16

Outpatient cases are from Ruebush et al. [60] and Krause et al. [30]. Inpatient cases are from White et al. [54] and Hatcher et al. [52].

**Table 2:** Diagnosis of babesiosis.

Epidemiology
– Residence in or travel to an area endemic for babesiosis
– *Ixodes* tick bite
– History of recent blood transfusion from a donor living or traveling in a *Babesia* endemic area

Symptoms

– Fever, fatigue, chills, sweats, headache, myalgia, anorexia, cough, arthralgia, nausea
– Less common: emotional lability and depression, hyperesthesia, sore throat, abdominal pain, conjunctival injection, photophobia, weight loss

Signs on physical examination

– Fever
– Splenomegaly, hepatomegaly, pallor

Common laboratory diagnostic procedures

– Identification of *Babesia* on Giemsa stained peripheral blood smears
– Amplification of *Babesia* DNA in blood using polymerase chain reaction
– Four-fold rise in *Babesia* antibody in acute or convalescent sera or identification of serum *Babesia* IgM antibody

**Table 3:** Treatment of babesiosis.

Treatment	Dose	Frequency
*Atovaquone and azithromycin*		
Atovaquone	Adult: 750 mg	Every 12 hours
	Child: 20 mg/kg	Every 12 hours
	(maximum 750 mg/dose)	
Azithromycin	Adult: 500 to 1000 mg	On day 1
	250 to 1000 mg	On subsequent days
	Child: 10 mg/kg	On day 1
	(maximum 500 mg/dose)	
	5 mg/kg	On subsequent days
	(maximum 250 mg/dose)	
*Clindamycin and quinine*		
Clindamycin	Adult: 600 mg	Every 8 hours
	Child: 7–10 mg/kg	Every 6–8 hours
	(maximum 600 mg/dose)	
	*Intravenous administration*	
	Adult: 300–600 mg	Every 6 hours
	Child: 7–10 mg/kg	Every 6–8 hours
	(maximum 600 mg/dose)	
Quinine	Adult: 650 mg	Every 6–8 hours
	Child: 8 mg/kg	Every 8 hours
	(maximum 650 mg/dose)	

All antibiotics are administered by mouth unless otherwise specified. All doses are administered for 7 to 10 days except for persistent relapsing infection (see text). For immunocompromised patients experiencing babesiosis, successful outcome has been reported using atovaquone combined with higher doses of azithromycin (600–1000 mg per day) [78]. Complete or partial exchange transfusion should be considered for treatment of severe babesiosis.
